# Sulfide stress tolerance as a controller of methane production in temperate wetlands

**DOI:** 10.1093/ismejo/wraf196

**Published:** 2025-08-28

**Authors:** Emily K Bechtold, Jared B Ellenbogen, Danhui Xin, Maricia Pacheco, Brandy M Toner, Yu-Ping Chin, William A Arnold, Sheel Bansal, Michael J Wilkins

**Affiliations:** Department of Soil and Crop Sciences, Colorado State University, Fort Collins, CO 80523, United States; Department of Soil and Crop Sciences, Colorado State University, Fort Collins, CO 80523, United States; Department of Civil, Construction, and Environmental Engineering, University of Delaware, Newark, DE 19716, United States; Southern California Coastal Water Research Project Authority, Chemistry Department, Costa Mesa, CA 92626, United States; Department of Soil, Water, and Climate, University of Minnesota, Minneapolis, MN 55455, United States; Department of Civil, Environmental, and Geo- Engineering, University of Minnesota, Minneapolis, MN 55455, United States; Department of Soil, Water, and Climate, University of Minnesota, Minneapolis, MN 55455, United States; Department of Civil, Construction, and Environmental Engineering, University of Delaware, Newark, DE 19716, United States; Department of Civil, Environmental, and Geo- Engineering, University of Minnesota, Minneapolis, MN 55455, United States; Northern Prairie Wildlife Research Center, U. S. Geological Survey, Jamestown, ND 5840, United States; Department of Soil and Crop Sciences, Colorado State University, Fort Collins, CO 80523, United States

**Keywords:** methane, methanogenesis, methylotrophic methanogenesis, sulfide toxicity, wetlands

## Abstract

Wetlands are a major source of methane emissions and contribute to the observed increase in atmospheric methane over the last 20 years. Methane production in wetlands is the final step of carbon decomposition performed by anaerobic archaea. Although hydrogen/carbon dioxide and acetate are the substrates most often attributed to methanogenesis, other substrates—such as methylated compounds—may additionally play important roles in driving methane production in wetland systems. Here we conducted mesocosm experiments combined with genome-resolved metatranscriptomics to investigate the impact of diverse methanogenic substrate amendment on methanogenesis in two high methane-emitting wetlands with distinct geochemistry, termed P7 and P8. Methanol amendment resulted in high methane production at both sites, whereas acetate and formate amendment only stimulated methanogenesis in P7 mesocosms, where aqueous sulfide concentrations were lower. In P7 sediments, formate amendment fueled acetogenic microbes that produced acetate, which was subsequently utilized by acetoclastic methanogens. In contrast to expression profiles in P7 mesocosms, active methylotrophic methanogen genomes from P8 showed increased expression of genes related to membrane remodeling and DNA damage repair, indicative of stress tolerance mechanisms to counter sulfide toxicity. Methylotrophic methanogenesis generates higher free energy yields than acetoclastic methanogenesis, which likely enables allocation of more energy toward stress responses. These findings contribute to the growing body of literature highlighting methylotrophic methanogenesis as an important methane production pathway in wetlands. By using less competitive substrates like methanol that provide greater energy yields, methylotrophic methanogens may invest in physiological strategies that provide competitive advantages across a range of environmental stresses.

## Introduction

Methane (CH_4_), a greenhouse gas with a warming potential ~28 times greater than carbon dioxide, has rapidly increased in concentration, highlighting its critical role in climate change dynamics [1–3]. Furthermore, due to its relatively short atmospheric lifetime, CH_4_ is a key target for short-term climate mitigation strategies. Wetlands are the largest natural sources of CH_4_ emissions worldwide and play a pivotal role in the recent increase of atmospheric CH_4_ over the last 20 years [4–6]. However, emissions from these ecosystems are highly variable and influenced by environmental factors that are not fully understood, including redox conditions, substrate availability, and microbial activity.

In wetland ecosystems, CH_4_ is primarily produced by methanogenic archaea through three distinct metabolic pathways: hydrogenotrophic (from H_2_/CO_2_ or formate), acetoclastic (from acetate), or methylotrophic (from methylated compounds) methanogenesis [7]. Hydrogenotrophic methanogenesis from H_2_/CO_2_ and acetoclastic methanogenesis are widely regarded as the most significant pathways driving CH_4_ emissions from wetlands. As such, they are the only pathways typically represented in process-based biogeochemical models [8]. However, recent studies demonstrate that methylotrophic methanogens play a crucial role in driving environmental CH_4_ emissions across diverse saturated ecosystems [9–15]. Additionally, although formate is a known electron donor in hydrogenotrophic methanogenesis, its role in supporting methanogenesis in wetland environments remains poorly characterized.

Methanogenesis has traditionally been associated with deeper sediment and soil layers, where the depletion of other electron acceptors removes thermodynamic constraints on this process. However, recent studies have revealed greater spatial variability in methane production and flux, with surficial wetland sediments identified as a potent source of methane flux [16–18]. These surface layers, especially under saturated conditions, rapidly become depleted of oxygen and receive abundant carbon inputs from surrounding vegetation and oxidative reactions involving reactive oxygen species (ROS) [19, 20]. Furthermore, although thermodynamic models often assume that redox conditions strictly constrain microbial metabolisms, the availability of noncompetitive substrates (such as certain C₁ compounds) may permit the simultaneous occurrence of sulfate reduction and methanogenesis, further challenging conventional redox paradigms [17, 21, 22]. Together, these biogeochemical conditions support robust microbial activity, yet the specific environmental drivers regulating substrate utilization—and consequently, methanogenesis—remain poorly understood.

The Prairie Pothole Region (PPR) of North America, one of the largest wetland complexes globally, offers a unique natural laboratory for exploring these biogeochemical processes. Comprising millions of small, mineral-soil depressional wetlands, the PPR functions as a biogeochemical hotspot [23, 24], with methane production shaped by a range of environmental variables relevant to other saturated soil systems. Previous studies have reported some of the highest terrestrial rates of sulfate reduction (20 μmol SO₄^2−^/cm^3^/day) and methane flux (>1 g CH₄/m^2^/day) in this region [17]. Despite these insights, a mechanistic understanding of the factors sustaining such biogeochemical activity remains incomplete. In particular, the role of low-molecular-weight carbon compounds (e.g. acetate, formate, methanol)—produced both abiotically and biotically—in supporting methanogenesis has not been fully characterized *in situ*.

Here, we used substrate-amended mesocosms to investigate how the availability of methanogenic substrates influences CH_4_ production in surficial sediments from two high-emitting PPR wetlands with distinct geochemical and microbial profiles. Using 16S rRNA gene community data, genome-resolved metatranscriptomes, metabolite analyses, and CH_4_ production measurements, we examined the contribution of different methanogenic pathways and substrates under varying geochemical conditions and investigated how methylotrophic methanogens adapt to environmental stress. These findings provide new insights into how methanogenic substrate availability and environmental stressors interact to shape CH₄ production in wetland sediments, with broader implications for understanding carbon cycling in redox-dynamic ecosystems.

## Materials and methods

### Field site and sample collection

The Cottonwood Lakes Study Area (47° 05’N: 99° 06’W) is a U.S. Fish and Wildlife Service Waterfowl Production Area and a long-term U.S. Geological Survey (USGS) research site located northwest of Jamestown, North Dakota. The site consists of 17 wetlands with varying degrees of water inundation, from temporary to permanent. These wetlands are classified along a gradient of lower to higher CH_4_ emissions [17, 23]. Samples were collected from two high-CH4-emitting wetlands, P7 and P8, which, despite being only 200 m apart and hydrologically connected, exhibit distinct geochemical characteristics. P7 functions as a flowthrough wetland characterized by low sulfide levels (<0.5 mM), lower ion concentrations including Mg (32.4 ± 6.9 mg/l), Na (11.8 ± 3.8 mg/l), and SO_4_ (111.7 ± 67.9 mg/l), and lower specific conductance (746 ± 147.2 μS/cm) [25]. In contrast, P8 is a discharge wetland that receives fluids rich in ions such as Mg (94.5 ± 66.3 mg/l), Na (68.5 ± 48.8 mg/l), and SO_4_ (358.6 ± 440.1 mg/l), yielding elevated specific conductance (1262.2 ± 710.3 μS/cm). Furthermore, wetland P8 pore fluids are rich in aqueous sulfide, with concentrations previously recorded up to 5 mM [17, 25, 26].

**Figure 1:**
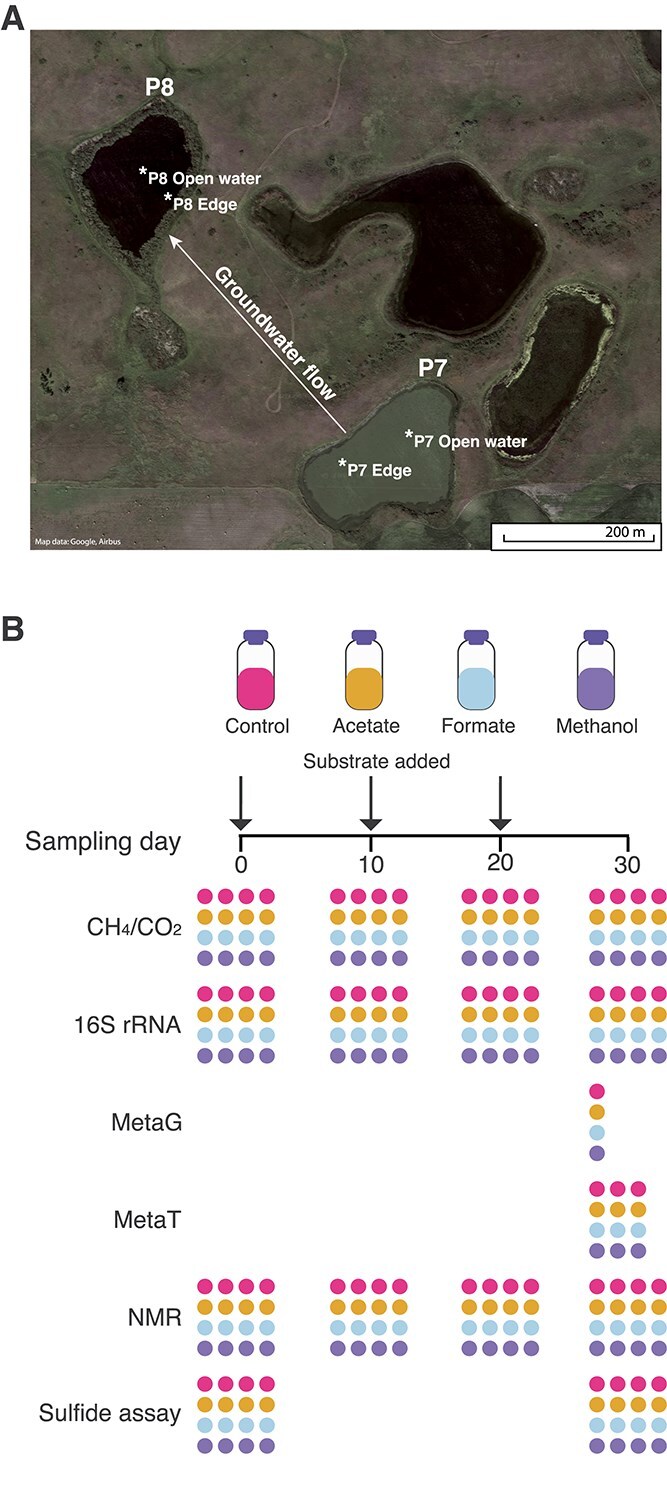
Mesocosm experiments investigating methane production in two hydrologically connected, high-emitting wetlands. (A) Experiments were conducted using materials from two distinct wetlands, P7 and P8, in the Prairie Pothole Region (PPR) in North America that are hydrologically connected via groundwater flow. Both wetlands are considered high methane-emitting wetlands. Each was sampled at two distinct internal ecosites, termed “open water” and “edge.” Edge denotes samples collected near the shore where the water column was shallower, whereas open water indicates the center of the wetland. Aerial images of were acquired from Google Earth (B) Mesocosms were set up to investigate the impact of field-relevant substrate dosing (acetate, formate, methanol) on methane production from within the different sites and ecosites (*n* = 64). Mesocosms were sampled for various analyses on the indicated days in the plot. NMR, nuclear magnetic resonance spectroscopy.

Samples were collected from two eco-zones within each wetland from the center, “Open Water,” of the wetland away from vegetation and nearer to the shoreline, “Edge,” where the water column was shallower (<2 ft) (Fig. 1A). Sediment samples from the sediment water interface were collected in October 2022 in triplicate using an Eckman dredge, and water samples were collected from the approximate center of the water column. Both were collected in sterile bottles, immediately placed on ice, and stored at 4°C until laboratory analysis.

### Mesocosm experimental setup and sample collection

To investigate the impact of methanogenic substrates on CH_4_-cycling, a laboratory mesocosm experiment was conducted. Each mesocosm contained 25 g of sediment and 25 ml of water from the respective wetland, sealed in sterile 160 ml serum bottles. The headspace in each bottle was flushed with N_2_ gas for 20 min to create anoxic conditions. Mesocosms were dosed with: acetate (acetoclastic condition), formate (hydrogenotrophic condition), methanol (methylotrophic condition), or nothing (unamended control). Four samples per treatment were prepared for each site and eco-zone, resulting in a total of 64 mesocosms (Fig. 1B).

Mesocosms underwent a 10-day pre-reduction period before the experiment began via incubation at 21°C in the dark. Substrates were added, and samples collected on Days 0, 10, 20, and 30 (Fig. 1B). All samples were collected with a low-gauge needle through a rubber butyl stopper to maintain anoxic conditions in the mesocosm. On each sampling day, headspace gas samples were collected to measure CH_4_ and CO_2_ production rates, and 2.5 ml of sediment was collected for use in DNA/RNA extractions, nuclear magnetic resonance spectroscopy (NMR), and sulfide assays. Following gas and sediment collection, additional methanogenic substrate was added; formate and methanol were added to a final concentration of 5 mM and acetate to 2.5 mM. Dosed mesocosms were then vortexed to ensure a homogenous slurry and flushed with filtered N_2_ gas for 20 min to reset headspace conditions, allowing for calculations of methane production rates for each time point. Mesocosms were kept in the dark at 21°C.

To collect time point measurements, mesocosm bottles were vortexed, and 10 ml of headspace gas was removed using a Hamilton gastight syringe through a rubber butyl stopper and simultaneously replaced with N_2_ gas. The gas was transferred to 6.9 ml Exetainers (Labco, Wales, UK) and stored at room temperature overnight. CH_4_ and CO_2_ were measured via gas chromatography (GC) (Agilent 8600 GC system) using a thermal conductivity detector (TCD) and a Porapak Q column. CH_4_ and CO_2_ concentrations for each 10-day period were calculated by comparing GC values to calibrated standard curves measured on the GC. Calculations were normalized to account for increased headspace volume resulting from sediment slurry removal at each time point and then normalized per gram of soil slurry in the mesocosm. Sediment–slurry samples were collected nondestructively and anoxically on sampling days, immediately after headspace collection. The 2.5 ml of sediment was aliquoted into three 2 ml tubes and immediately stored at −80°C.

### DNA/RNA extraction

DNA was extracted from every sample and time point using the Zymo Quick-DNA Fecal/Soil Kit (Zymo Research, CA, USA) following the manufacturer’s directions. RNA was extracted from 24 samples, including triplicate samples from Day 30 of each treatment from the open-water ecosite of P7 and P8 using the Zymo Quick-RNA Fecal Soil Kit with the optional DNase treatment step.

### 16S rRNA gene sequencing

DNA samples were amplified using barcoded primers for the V4-V5 regions of the 16S rRNA gene, following Earth Microbiome Project protocols, with forward primer 515F [27] and reverse primer 806R [28]. Libraries were normalized using the SequalPrep Normalization Kit (ThermoFisher, Waltham MA) and pooled for sequencing on the MiSeq System (Illumina) at the University of Colorado-Boulder, using the v2-500 cycle kit and 251 bp paired-end sequencing.

QIIME2 (v. 2021.2) [29] was used to demultiplex, pair, and trim paired-end reads, with amplicon sequence variants (ASVs) determined using DADA2 [30]. Taxonomy was assigned using the naïve Bayes sklearn classifier trained with the GTDB-Tk (release 207) database [31]. Sample reads ranged from 3515 to 44 358 with samples containing fewer than 10 000 reads excluded (5 of 256 samples).

### Metagenomic sequencing

Metagenomes were obtained from a subset of nine samples collected at the end of the experimental period (Supplemental Table S1). These metagenomes were sequenced to construct a metagenome-assembled genome (MAG) database for mapping metatranscriptomic data. Library preparation was performed using Ovation Ultralow System V2 (Tecan), and sequencing was conducted on a NovaSeq 6000 System (S4 flow cell, 2 × 150 bp) (Illumina) at the Genomics Shared Resource Facility at the University of Colorado Anschutz. Raw reads were trimmed using Sickle (pe) [32], and the outputs were assembled using Megahit(v1.2.9) [33] with parameters --k-min 31 --k max 121 --k-step 10(v1.2.9) [34]. MAG quality was determined using CheckM [35], with only medium- and high-quality (MQ/HQ) MAGs retained (completeness ≥50% and contamination <0%) [36]. All samples then underwent iterative assembly in which trimmed reads were mapped against the MQ/HQ MAGs; reads that did not map were assembled and binned as described. Unmapped reads were then combined from metagenomes within the same site and co-assembled using the pipeline described above. These bins were then dereplicated at 99% identity using dRep (v3.0.0) [37], taxonomy was assigned using the Genome Taxonomy Database Toolkit (GTDB-tk v2.3.0 r207) [31], and MAGs were annotated using Distilled and Refined Annotation of Metabolism (DRAM) (v1.4.4) [38]. These MAGs were combined with the Multiomics for Understanding Climate Change (MUCC v2.0.0) database totaling 3634 MQ/HQ dereplicated MAGs found across freshwater wetlands [39].

### Metatranscriptomic sequencing

RNA libraries were prepared with ribosomal depletion and messenger RNA enrichment using the Zymo-Seq RiboFree Total RNA Library Kit (Zymo Research, CA, USA). Libraries were sequenced on the NovaSeq6000 Platform (S4 flow cell, 2 × 151 bp) (Illumina) at the Genomics Shared Resource Facility at the University of Colorado Anschutz (Supplemental Table S2). Metatranscriptomic reads were trimmed using BBDuk (k = 23 mink = 11 hdist = 1 qtrim = rl trimq = 20 minlength = 75) [40]. Trimmed reads were then mapped to the MAG database using Bowtie2 (-D 10 -R 2 -N 0 -L 22 -I S,0,2.50) [41]. The resulting SAM files were converted to BAM files using samtools and were then filtered using reformat.sh scripts (using flags idfilter = 0.97 pairedonly = t primaryonly = t) as implemented in bbtools. Transcripts were then counted using htseq-count (v0.013.5) (-a 0 -t CDS -I ID –strnaded = reverse) [42]. Any read with <5 counts was removed, and counts were subsequently transformed to geTMM (gene length corrected trimmed mean of M-values) in R [43]. Using previously established methods [44], the median number of genes that had geTMM values greater than 0 in a MAG was 4; therefore, MAGs with fewer than four genes with counts were determined to be inactive and those genes were filtered out. Additionally, genes needed to be found in two-thirds of samples within a treatment to be considered as actively transcribed.

### Assigning methanogenic pathway utilization to metagenome-assembled genomes

MAGs were classified as methanogens if they encoded genes of the methyl coenzyme reductase (*mcrABG)* and/or heterodisulfide reductase (*hdrabcde)* complexes [39]. These were then further assigned a specific methanogenic substrate-use potential following previously published rules [9].

### Differential gene expression analysis

Differential expression of genes between treatment and sites was determined using MaAsLin2 [45] with significance determined based on the MaAsLin2 criteria, if *q* < 0.25, following a Benjamini–Hochberg *P*-value correction. MaAsLin2 analyses compared (i) different sites based on substrate amendment or (ii) within a site, each substrate amendment was compared to the control. Only genes present in at least two samples per treatment and annotated with Kyoto Encyclopedia of Genes and Genomes (KEGG) Orthology (KO) were considered. Additionally, MaAsLin2 was used to compare the activity of *Methanmethylovorans* in the methanol-amended treatment between P7 and P8 sediments. Genes with an average geTMM >1 were retained for analysis, and identities were determined using DRAM annotations or BLASTx (bitscore >60, percent identity >30). Results were visualized using ggplot2 in R by plotting log₁₀(*q* value) against the MaAsLin2 coefficient.

### Nuclear magnetic resonance spectroscopy

Concentrations of acetate, formate, and methanol were determined in all samples using ^1^H NMR. Sediment supernatant (450 ml) was combined with 50 μl 2,2-dimethyl-2-silapentane-5-sulfonate- d_6_ (DSS-d_6_) in D_2_O (20 μl, 5 mM) and transferred into 5 mm NMR tubes. A Bruker NEO400 with a 400 MHz frequency equipped with a Broadband Frequency Observation smart probe regulated at 298K at the Analytical Resource Core Magnetic Resonance Laboratory at Colorado State University. The 1D ^1^H spectra were acquired with a single pulse sequence with a spectral width of 15.622 ppm and 32 transients, an acquisition time of 2.62 s, and a relaxation delay of 15 s. Chemical shifts were referenced to the ^1^H methyl signal in DSS-d_6_ at 0 ppm. Spectra were manually processed, and metabolite identification was assigned and quantified using MestReNova 15. Quantification was based on fitted metabolite signals relative to the internal standard (DSS-d_6_).

### Sulfide measurements

Sulfide concentration in the mesocosms was measured using the HACH 8131 methylene blue method. Supernatant (10–100 μl) was combined with distilled water to a final volume of 1 ml, followed by the addition of 40 μl of HACH Sulfide Reagent 1 and Sulfide Reagent 2. After 5-min, absorbance was measured at 665 nm, and sulfide concentration was determined using a standard curve.

### Sulfide toxicity tests

To test whether sulfide toxicity affects acetoclastic, but not methylotrophic, methanogens, further anoxic mesocosms were established using sediment from P7, which has lower sulfide levels and more active acetoclastic methanogens compared to P8. Six bottles per substrate and unamended controls (18 bottles total) were prepared by combining 10 g of sediment with 10 ml of water from P7, sealing, and flushing the headspace with N_2_ for 20 min. Mesocosms were either unamended or amended every 7 days with 5 mM methanol or 2.5 mM acetate. On sampling days, headspace was collected to measure CH_4_ and CO_2_ production, and 2 ml of sediment slurry was removed to measure sulfide. On Days 14, 21, and 28, sulfide was added to three mesocosms per treatment in increasing concentrations over time at 1.5, 2.5, and 4.5 mM, respectively. These values were chosen to mimic values previously measured *in situ* in P8, which reached up to 5 mM [17]. Sulfide levels were measured to ensure the desired concentrations were achieved.

### Statistical analyses

Bray–Curtis dissimilarity was calculated for the entire microbial and methanogen communities using the vegan package [46] in R [47]. Differences between sites, treatments, and times were assessed using permutational multivariate analysis of variance (PERMANOVA) implemented using the adonis2 function (vegan) and visualized using nonmetric multidimensional scaling (NMDS) in ggplot2 [48]. Additionally, analysis of variance (ANOVA) followed by Tukey’s Honestly Significant Difference (HSD) post hoc analysis to determine pairwise differences was performed in R on all univariate data produced from the GC, NMR, and sulfide analyses to determine the differences over time and between treatments.

## Results

### Microbial community differences between wetlands are greater than within wetland differences

Wetland P7, characterized by relatively low concentrations of sulfide (~0.5 mM) and other ions, and wetland P8, with high concentrations of sulfide (~5 mM) and other ions [17], hosted distinct *in situ* sediment methanogen communities. All three methanogenic pathways were present in relatively similar proportions in P7 sediments, whereas P8 samples were dominated by methylotrophic methanogens and lacked detectable acetoclastic methanogens (Fig. S1). Additionally, prior to substrate amendment, mesocosm microbial communities from different eco-zones within the same wetland were compositionally more similar to each other than to communities from the other wetland, reflecting site-specific microbial signatures seen *in situ* (Fig. S2). PERMANOVA of these samples showed that site explained 49.6% of the variability (Pseudo-*F* Statistic = 82.0, *P* < .001) whereas eco-zone explained only 7.4% of the variability (Pseudo-*F* Statistic = 12.3, *P* < .001). Additionally, eco-zones within the same wetland exhibited similar microbial community responses to substrate amendments over time (Fig. S2). However, CH_4_ production was consistently higher in sediment mesocosms from open waters compared to those constructed from edge sediments (P7: *P* = .04; P8: *P* = .001). Even though CH_4_ production and flux are spatially heterogeneous within individual wetlands [24], we observed similar overall trends across sites. For example, in P7, all three substrates resulted in increased CH_4_ production compared to the control in both ecosites (Fig. S2E and F). In contrast, in P8, formate and acetate had a lower impact on methanogenesis in both ecosites (Fig. S2G and H). Despite similarities in community composition between eco-zones in a given wetland, open water mesocosms consistently generated more CH_4_ over the course of the incubation period. As such, we focused metatranscriptomic analyses on communities from these incubations.

### Methanogenic substrates differentially impact CH_4_ production and sediment chemistry

**Figure 2:**
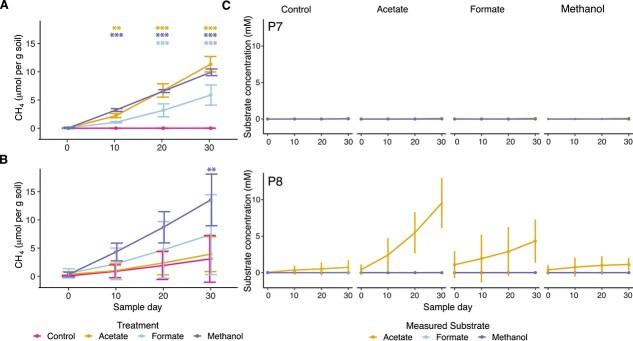
Methane production and substrate utilization in mesocosms from each site over time. Mesocosms contained sediment and water from either the P7 or P8 wetland in the Prairie Pothole Region of North America. (A) Dosing with all three substrates, methanol, acetate and formate, promoted increased methanogenesis in P7 mesocosms, indicated by significantly higher cumulative dissolved methane concentrations (μmol CH_4_/g soil) than in the control mesocosms. Mesocosms contained sediment and water from either the P7 or P8 wetland in the Prairie Pothole Region of North America. (B) Only methanol addition promoted greater methanogenesis in P8. Each time point represents cumulative CH_4_ generation. Asterisks indicate significant differences in μmol CH_4_ concentrations between substrate and controls determined using analysis of variance (ANOVA) followed by Tukey HSD where ^*^, *P* ≤ .05; ^**^, *P* ≤ .01; ^***^, *P* ≤ .001. (C, D) Acetate accumulated over time in the acetate- and formate-amended mesocosms using P8 sediments only. Accumulation of formate or methanol was not observed under any experimental condition. Plots represent each mesocosm, and lines represent substrate concentration within each mesocosm measured using nuclear magnetic resonance (NMR) spectroscopy.

Dosing mesocosms with acetate, formate, or methanol differentially impacted CH_4_ production in sediment mesocosms from P7 and P8 (Fig. 2A and B). Methanol was the only substrate that significantly stimulated CH_4_ production above background levels in P8 sediments (methanol: *P* < .001; acetate *P* = .98; formate: *P* = .20), whereas the addition of each substrate to P7 sediments resulted in increased CH_4_ production compared to the unamended controls (methanol: *P* < .001; acetate: *P* < .001; formate: *P* < .001). Within the P7 mesocosms, acetate amendment yielded the most CH_4_/g soil, whereas formate had the lowest CH_4_/g soil, and methanol was intermediate. In contrast, acetate amendment in P8 did not yield more CH_4_/g soil than the unamended mesocosms. Additionally, there was no significant difference in CH_4_ production between P7 and P8 in formate-amended mesocosms (Fig. S3).

We measured levels of added substrates across the duration of the experiment (Fig. 2C) and quantified aqueous sulfide at the initial and final time points (Fig. S4). Within the P8 mesocosms, acetate accumulated in the acetate- and formate-amended sediments but not in any other treatments. Further, neither formate nor methanol was found to accumulate in any mesocosm. Sulfide concentrations were initially higher in P8 than in P7 (*P* < .001) and remained higher at the end of the experiment. Moreover, sulfide levels increased over time in all mesocosms except the methanol amendment (Fig. S4 and Supplemental Data S3).

### Methanogen presence and activity vary in response to substrate amendment

**Figure 3:**
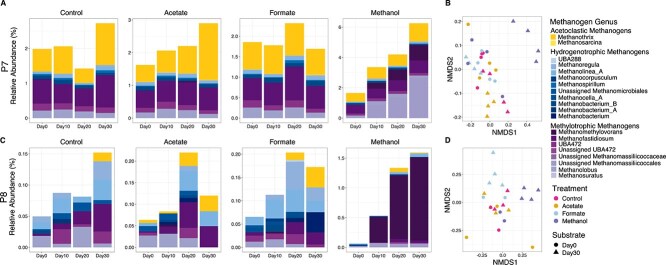
Changes in community composition between site and in response to substrate amendment over time. 16S rRNA gene data were collected from mesocosms every 10 days over a 30-day period. (A) Profiles of the relative abundance of the methanogen communities from substrate-amended mesocosms made using P7 sediment shows that acetoclastic methanogens increase over time in response to acetate amendment, no change is observed in the methanogen community in response to formate, and methylotrophs increase in response to methanol. (B) Corresponding nonmetric multidimensional scaling (NMDS) of the methanogen community shows that methanol has a greater impact on methanogen community structure over the course of the experiment than acetate or formate. (C) P8 had a lower relative abundance of methanogens than P7 and showed different responses to substrate amendment. Acetate and formate amendment did not enrich any methanogens in the P8 in relation to the control community but amendment with methanol did result in an increased abundance of *Methanomethylovorans*—a methylotrophic methanogen. (D) The corresponding NMDS of the methanogen community in P8 shows that acetate amendment had no impact on community structure, whereas methanol amendment had the greatest impact.

Microbial community structure and composition were assessed over time using 16S rRNA gene sequencing. Methanogen communities were distinct between the two wetlands at both the start (PERMANOVA: *R*^2^ *=* 0.52, Pseudo-*F* Statistic = 30.9; *P* < .001) and end (PERMANOVA, *R*^2^ = 0.38, Pseudo-*F* Statistic = 40.4; *P* < .001) of the experiment (Fig. 3), with P7 maintaining a higher total relative abundance of methanogens throughout the incubation period compared to P8. Acetate and formate amendments resulted in relatively minimal changes in methanogen community structure for both P7 and P8, whereas methanol amendment yielded a significant enrichment of methylotrophic methanogens, especially in P8. In P7, both *Methanolobus* and *Methanomethylovorans* were enriched, although only *Methanomethylovorans* showed significant enrichment in P8. By the end of the experiment, the relative abundances of *Methanomethylovorans* were similar between each site in methanol-amended mesocosms.

**Figure 4:**
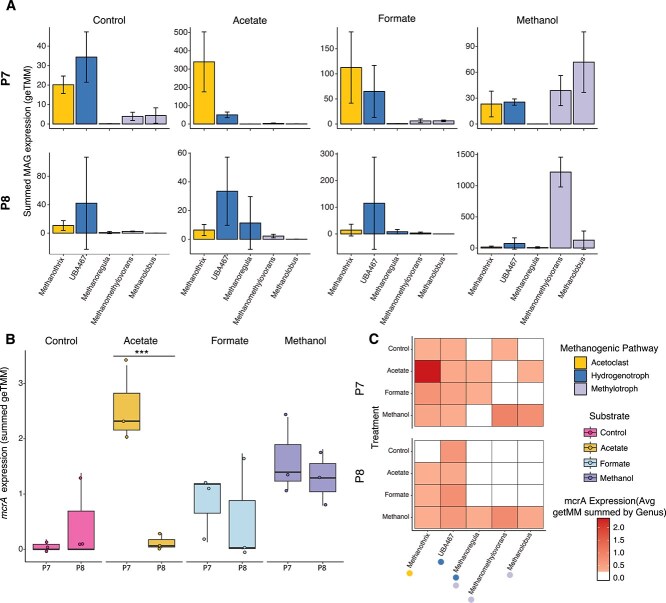
Total activity of methanogens and *mcrA* across sites and substrates. Acetate, formate, and methanol substrate amendments had unique impacts on expression of methanogens and methanogenesis genes (*n* = 24 metatranscriptomes). (A) Summed total expression of metagenome-assembled genomes (MAGs) at the genus level using geTMM normalization revealed that methanogens were differentially impacted in P7 and P8. In methanol-amended mesocosms, *Methanomethylovrans* activity increased but showed vastly different levels of expression between sites. (B) Total *mcrA* expression across treatments demonstrated that acetoclastic methanogenesis in P7 had higher expression compared to P8, with no other treatments showing significant differences in marker gene expression between wetlands. The boxes represent the interquartile range with the median *mcrA* geTMM (gene length–corrected trimmed mean of M-values) expression value of each site given by the black line within each box. The upper and lower quartiles are shown based on the lines extending from each box, whereas individual points represent outliers defined based on ggplot2 default parameters. Relationships between sites were analyzed for significance using an analysis of variance followed by a Tukey’s Honest Significance Test to perform pairwise comparisons indicated by asterisks. (C) *mcrA* expression by summed genus revealed that *Methanomethylovrans* exhibited similar expression in methanol-amended mesocosms between sites, despite differences in total expression.

To examine microbial activity in response to the substrate amendments, we performed genome-resolved metatranscriptomic analyses (Fig. 4). We recovered MAGs from 158 Gbp of metagenomic data derived from mesocosms and 171 Gbp of metagenomic data derived from Prairie Pothole field sediment samples, resulting in 314 unique medium- and high-quality MAGs [36]. These data are included in the previously published MUCC database v 2.0.0 [39, 49]. We mapped metatranscriptomic reads to the entire MUCC v2.0.0 database, which comprises 3634 unique MAGs. Transcripts were recruited by 490 of these MAGs.

We analyzed the total gene activity within each MAG by summing the geTMM values and averaging across MAGs within the same genus (Fig. 4A). CH_4_ production across treatments was predominantly attributed to five methanogen genera (Fig. 4A). Acetoclastic methanogenesis was driven by *Methanothrix*, hydrogenotrophic methanogenesis by *UBA467* and *Methanoregula*, and methylotrophic methanogenesis by *Methanolobus* and *Methanomethylovorans*. In P7, *Methanothrix* displayed high activity in acetate-amended mesocosms and increased activity in formate-amended mesocosms. However, *Methanothrix* showed consistently low activity across all P8 mesocosms. Furthermore, *Methanolobus* showed similar increases in overall activity in methanol-amended mesocosms in both P7 and P8. In contrast, the highest total gene expression was attributed to *Methanomethylovorans* in P8 methanol-amended mesocosms, exhibiting 9.7-fold higher activity compared to the same genus in P7 and 3.6-fold higher activity than any other methanogen genus across all treatments and sites.

At the individual gene level, summed *mcrA* expression exhibited trends that closely mirrored CH_4_ production data, in contrast to trends observed with summed MAG activity (Fig. 4B). Acetate-amended P7 mesocosms showed the highest *mcrA* expression, which was significantly higher than in P8 mesocosms that received exogenous acetate (*P* = .005). However, no wetland-specific differences in *mcrA* expression were observed for unamended control, formate-amended, or methanol-amended mesocosms. To further investigate these trends, we analyzed *mcrA* expression within each genus (Fig. 4C). *Methanomethylovorans* exhibited similar expression in P7 and P8 and displayed the highest *mcrA* expression in methanol-amended mesocosms, confirming its role as the dominant genus driving CH_4_ production in this treatment.

Formate amendment stimulated both hydrogenotrophic and acetoclastic methanogenesis in P7 (Fig. 4A). There was a slight increase in formate dehydrogenase expression in *UBA467* in P7 but not P8; however, its expression remained low (Fig. S5A). Although formate amendment resulted in production of acetate in P8 (Fig. 2C), no acetate accumulated in P7. However, increased expression of *Methanothrix* genes, including acetyl-CoA synthetase, was observed, suggesting indirect cross-feeding of acetoclastic methanogens (Fig. S5B).

### Substrates differentially shape community function and metabolism across sites

**Figure 5:**
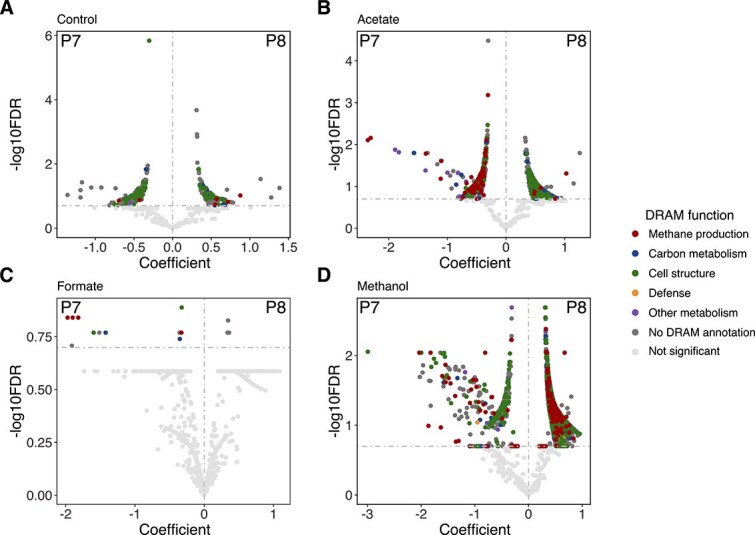
Differential expression of genes compared between sites for each substrate amendment. Differentially expressed genes, determined using MaAsLin2, were compared between sites for (A) unamended control mesocosms, (B) acetate-amended mesocosms, (C) formate-amended mesocosms, and (D) methanol-amended mesocosms. Genes were annotated with DRAM and are colored based on modules provided in DRAM. The unamended controls showed some site-specific differences in genes related to cell structure such as various housekeeping genes. The biggest difference between wetlands in expression between the two sites was observed in the acetate and methanol treatments. Many of the higher expressed genes in the acetate treatment in P7 were related to methanogenesis. In methanol-amended mesocosms, both wetlands had a high abundance of different methanogenesis genes, but P8 also had a much higher number of differentially expressed genes, many of which were involved in housekeeping functions (cell structure) and various metabolic processes. The *x*-axis represents the effect size and direction of association. The *y*-axis represents the −log10 of the *q*-value where significant differences were determined when *q* < 0.2. Vertical and horizontal dashed lines indicate P7 (left) vs P8 (right) and significant (above) vs nonsignificant (below) DRAM results (respectively).

To understand how overall gene expression varied between wetlands and in response to substrate amendment, we identified differentially expressed genes between treatments, retaining only those with KO annotations (Fig. 5) (Supplemental Data S1). In the unamended mesocosms, 311 genes were differentially expressed between P7 and P8 sediments, with 185 genes upregulated in P7 and 126 genes upregulated in P8. Of these, 109 genes were functionally annotated. Both sites exhibited a similar number of differentially expressed housekeeping genes. However, site-specific differences were observed: P7 showed an increase in the expression of transporter genes (37 out of 46 differentially expressed genes) and sulfur metabolism genes (8 out of 8), whereas P8 showed an increase in gene expression related to central carbon metabolism (12 out of 16) and C1 metabolism (12 out of 18) (Fig. 5A).

In the acetate-amended treatment, 819 genes were differentially expressed between P7 and P8 incubations (Fig. 5B). The majority (495 genes) showed increased expression in P7, including a substantial enrichment of methanogenesis genes. Specifically, 66 of the 70 differentially expressed methanogenesis genes increased expression in P7 (Fig. 5B). In contrast to other amendments, only 16 genes showed differential expression in the formate-amended mesocosms, all of which had increased expression in P7, including four associated with methanogenesis (Fig. 5C). Finally, methanol amendment had the greatest impact in differentially expressed genes between P7 and P8 sediments; 7157 genes were differentially expressed with only 502 showing increased expression in P7, whereas 6655 showed increased expression in P8. Of 2290 differentially expressed genes that could be annotated, 797 were housekeeping genes, 331 were involved in amino acid biosynthesis, 258 in C1 metabolism, 274 in central carbon metabolism, 218 in CH_4_ production, 87 in the electron transport chain, and 81 in pyruvate metabolism (Fig. 5D).

In addition to comparing expression between sites, we analyzed differential expression within each site by comparing each treatment to the unamended control (Figs S6 and S7). For P7 mesocosms, all treatments showed increased expression of CH_4_ production genes, with the acetate treatment exhibiting the highest number of differentially expressed genes. In contrast, P8 displayed amendment-specific trends: the acetate treatment had very few differentially expressed genes with none related to CH_4_ production, and the formate treatment showed no differential expression. In contrast, the methanol treatment had several thousand differentially expressed genes, many of which were associated with CH_4_ production.

### Sulfide inhibits acetoclastic methanogenesis

**Figure 6:**
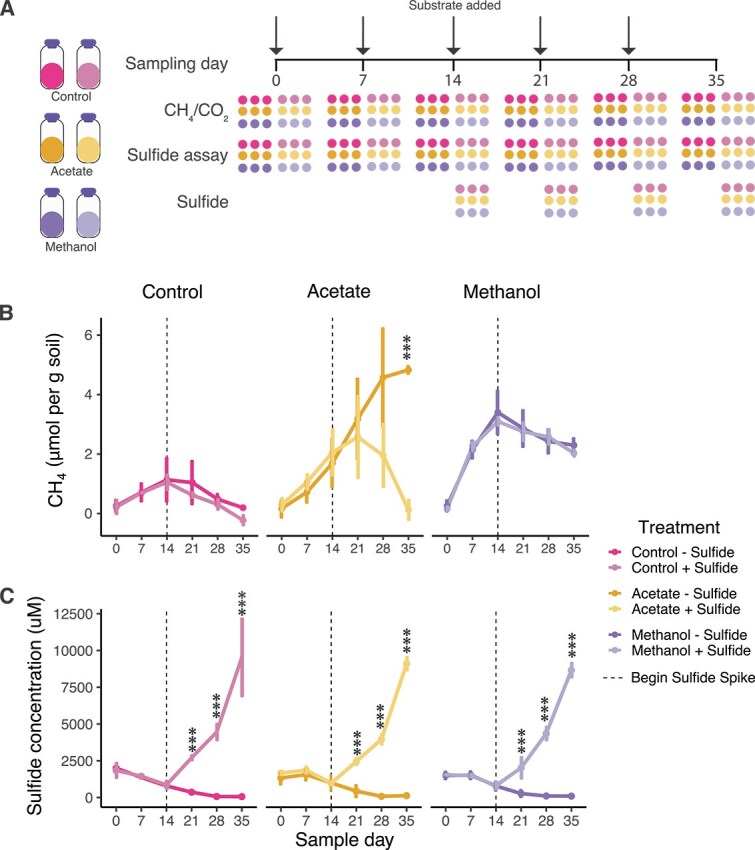
Experimental design for mesocosm experiments testing whether sulfide addition to P7 soil inhibits methanogenesis. (A) Sulfide toxicity was tested using P7 sediment in which increasing concentrations of sulfide were added (lighter-shaded circles) to the mesocosms after 14 days, when they were showing clear signs of methane production (*n* = 18). (B) Sulfide significantly inhibited methanogenesis in acetate-amended mesocosms but not in methanol-amended mesocosms, demonstrating that acetoclastic methanogens are susceptible to sulfide toxicity. (C) Sulfide levels uniformly increased over the experimental period following addition on Day 14.

Given the low activity of acetoclastic methanogens and high activity of methylotrophic methanogens in sulfide-rich P8 mesocosms (Fig. 4), we hypothesized that high sulfide concentrations were inhibiting acetoclastic but not methylotrophic methanogenesis. To test this, we established a secondary mesocosm experiment where we exposed P7 sediments to elevated sulfide concentrations characteristic of P8 (Fig. 6A). Mesocosms were amended with either no added substrate, acetate, or methanol, and starting on Day 14—after methanogenesis had been initiated in the treatments—they were subsequently dosed with sulfide weekly for the remainder of the incubation period.

In acetate-amended mesocosms, CH₄ production declined progressively following the initial sulfide addition: a 22% reduction was observed on Day 21 compared to the no-sulfide control, a 59% reduction on Day 28, and a 96% reduction by Day 35. This decline was statistically significant over the course of the experiment (ANOVA, *P* = .03), with a highly significant difference by Day 35 (*P* < .001) (Fig. 6B). In contrast, sulfide addition had no significant effect on CH₄ production in methanol-amended mesocosms (ANOVA, *P* = .53), with only a 3% difference in CH₄ production observed on Day 35 between sulfide-treated and untreated mesocosms (Fig. 6B). These results support our hypothesis that high sulfide concentrations selectively inhibit acetoclastic, but not methylotrophic, methanogenesis.

### Methylotrophic methanogens increase expression of stress response genes under high sulfide conditions

**Figure 7:**
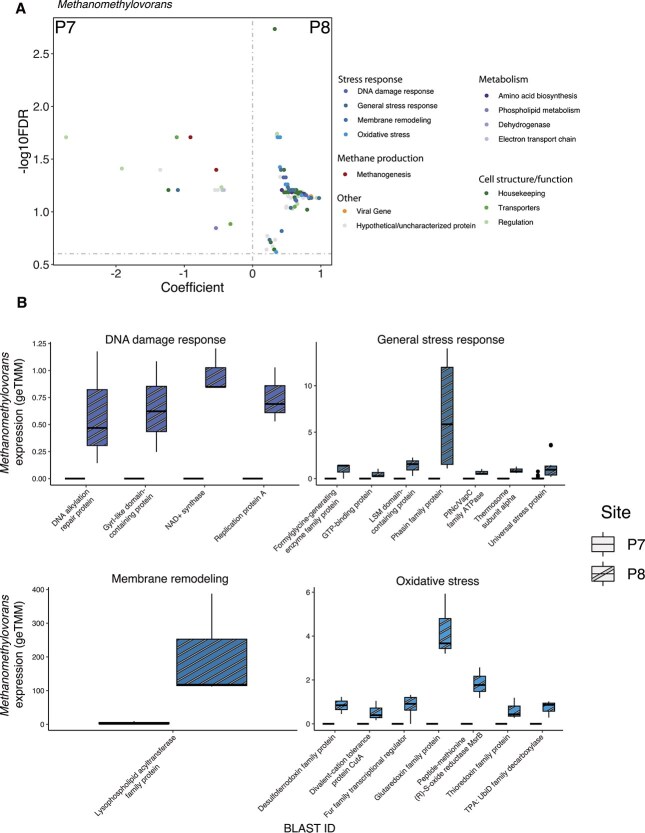
Differential expression analysis of *Methanomethylovorans* genes between sites. (A) Using MaAsLin2, several stress response genes showed increased expression in P8 (right of dashed line) compared to P7 (left). To capture the most highly transcribed genes, only those with a geTMM value above 1 were retained. Significant differences were determined when *q* < 0.25. (B) All genes with significant differential expression in the stress tolerance categories were plotted. This analysis revealed that a single gene involved in membrane remodeling was the largest contributor to the difference in the total expression of *Methanomethylovorans* between the sites. The boxes represent the interquartile range with the median geTMM (gene length corrected trimmed mean of M-values) gene expression of each site given by the black line within each box. The upper and lower quartiles are shown based on the lines extending from each box whereas individual points represent outliers defined based on ggplot2 default parameters. Boxes displaying P7 data are too small to see in all cases.

*Methanomethylovorans* was the most active methanogen in methanol-amended mesocosms in both P7 and P8 (Fig. 4). Despite similar levels of CH_4_ production and *mcrA* expression between these mesocosms, *Methanomethylovorans* exhibited significantly higher total gene expression in P8 than in P7 (Fig. 4A). To identify the differentially expressed genes that accounted for this discrepancy, we conducted MaAsLin2 analysis on expressed genes associated with the active *Methanomethylovorans* MAGs, comparing expression between the two sites (Fig. 7A). We focused on the most dominantly expressed genes, limiting our analysis to those with a geTMM value of 1 or higher. For genes without functional annotations, we performed BLASTx searches and categorized them into four functional groups: Stress Response, Cell Structure/Function, Metabolism, or Other (Supplemental Table S2).

Of the 4698 *Methanomethylovorans* genes that recruited transcripts in methanol-amended mesocosms, 152 genes reached our expression threshold and were included in downstream analyses. Of these, 124 genes were differentially expressed between methanol-amended P7 and P8 mesocosms. A total of 99 showed increased expression in P8 compared to only 15 in P7. In P8, 24 stress response genes, 36 cell structure/function genes, and 8 metabolism-related genes showed increased expression. Interestingly, among the three differentially expressed methanogenesis genes, two showed significantly higher expression in P7.

The stress response genes were categorized into four subgroups: DNA Damage Response, General Stress Response, Membrane Remodeling, and Oxidative Stress (Fig. 7B). The Membrane Remodeling category was composed of a single gene—lysophospholipid acyltransferase family protein—which exhibited the highest expression among all genes analyzed. This gene was a major contributor to the observed disparity in total *Methanomethylovorans* gene expression between methanol amended P7 and P8 mesocosms.

To better understand the potential for other methanogens to cope with sulfide stress, all methanogens genomes were examined for the presence of sulfur assimilation and dissimilation genes [50]. None of the methanogens encoded genes associated with dissimilatory sulfate reduction. However, *Methanomethylovorans* contained genes for a sulfate transporter (*cysA*), assimilatory phosphoadenosine phosphosulfate (PAPS) reductase (*cysH*), and a ferredoxin-dependent sulfite reductase (*fsr*). *Methanolobus* also encoded *cysA* and *cysH*, along with serine acetyltransferase (*cysE*), and cysteine synthase (*cysK*), indicating potential for cysteine biosynthesis via assimilatory sulfur reduction. However, *Methanothrix* only encoded the sulfate transporter gene (*cysA*) and lacked other key sulfur assimilation genes. Although *fsr* was not expressed, *cysH* had significantly higher expression in *Methanomethylovorans* in P8 methanol treatment compared to all other sites and treatments and compared to *Methanolobus* (*P* < .001) (Fig. S8). Additionally, *cysE* and *cysK* showed low expression levels in the methanol-amended treatments in both P7 and P8 but were not significantly different from the other treatments (Fig. S9).

## Discussion

Accurately predicting CH_4_ flux from wetlands has historically been challenging due to incomplete knowledge of CH_4_ cycling in wetlands and the difficulties in incorporating complex ecosystem interactions and microbial metabolisms into process-scale biogeochemical models [4, 8, 51, 52]. Here, we focused on understanding how sediments from two historically high CH_4_-emitting Prairie Pothole wetlands respond to diverse substrates utilized across methanogenic metabolisms. Despite wetlands P7 and P8 being hydrologically connected, sediment mesocosms showed distinct microbial metabolic responses to substrate amendment. This suggests that no single mechanism drives high CH_4_ production, even within a single ecosystem and highlights the complexities of developing accurate CH_4_ models [51]. Canonically, acetoclastic and hydrogenotrophic methanogenesis are considered the dominant pathways in most anoxic environments, including freshwater wetlands [53–56], whereas methylotrophic methanogenesis is often overlooked and therefore not included in process-scale biogeochemical models [8, 9]. Our findings challenge this convention, as methylotrophic methanogenesis not only contributed significantly to CH_4_ production in both P7 and P8 but was the primary pathway driving CH_4_ production in P8 mesocosms based on measured CH_4_ production and metatranscriptome results. Previous work has reported high concentrations of both methanol and CH_4_ in porewaters at the sediment water interface of these wetlands [17]. Given that methanol can be a noncompetitive substrate [21, 57], it is likely that these substrate pools serve as a key driver of methanogenic activity in some Prairie Pothole wetlands.

Formate is as an alternative to H₂ in hydrogenotrophic methanogenesis alongside CO₂, facilitated by the enzyme formate dehydrogenase [58]. In Prairie Pothole wetlands, formate can be produced through a wide range of processes including fermentation, release through plant roots, or through ROS-mediated reactions and could therefore support methanogenesis within this system [19]. However, among the three substrates tested, formate amendment resulted in the lowest CH_4_ production rates. Formate dehydrogenase expression showed a slight increase in the formate-amended mesocosms; however, these changes were only significant in P7 in comparison with the unamended mesocosms. This suggests that methanogens were directly utilizing formate for methanogenesis, albeit in small amounts. Additionally, formate can be metabolized by acetogenic bacteria, leading to acetate production [59]. Reflecting biogeochemical differences between wetland P7 and P8 sediments, we observed differing responses to formate amendment. Supporting the absence of acetoclastic methanogenesis in P8 mesocosms, we measured the accumulation of acetate likely associated with the activity of formate-metabolizing acetogenic bacteria. In contrast, acetate production in response to formate amendment in P7 mesocosms stimulated the activity of the acetoclastic methanogen, *Methanothrix*. Even though formate directly and indirectly stimulates methanogenesis in these wetland sediments, these data suggest that these processes are unlikely to be a primary driver of CH_4_ production in PPR wetlands.

Consistent with previous studies, acetate amendment resulted in the highest CH_4_ production levels in P7, which was exclusively driven by members of the genus *Methanothrix*. In contrast, acetate had no significant impact on CH_4_ production in P8. Because methanogenesis is one of the least energetically favorable metabolic processes, substrates can be preferentially used as electron donors in other respiratory metabolisms, such as sulfate reduction, when alternative electron acceptors are available. Given previous research demonstrating that sulfate reduction rates in P8 sediments are some of the highest ever recorded in a terrestrial ecosystem [17], we hypothesized that acetate amended to P8 mesocosms was consumed by non-methanogenic microbes, resulting in low CH_4_ production. To test this, we measured acetate, formate, and methanol concentrations over the course of the experiment and found that formate and methanol were consumed, whereas acetate concentrations increased in P8 mesocosms but not in P7. Supporting these geochemical observations, *mcrA* had significantly higher expression in acetoclastic methanogens in P7 compared to P8. This indicates that although acetate was available for methanogens in P8, acetoclastic methanogenesis was inhibited.

High sulfide levels have been implicated in inhibition of acetoclastic methanogenesis in salt ponds [60]. Given that P8, both historically and in this study, exhibits unusually high sulfide concentrations for a freshwater wetland—with *in situ* levels reported up to 5 mM [17]—we tested whether sulfide accumulation specifically inhibits acetoclastic methanogenesis while leaving methylotrophic methanogenesis unaffected. To do this, we used sediment from wetland P7, where lower aqueous pore water sulfide concentrations (~0.5 mM) have been previously reported. Sulfide bioassay incubations revealed that sulfide amendment had an immediate impact on acetoclastic methanogenesis and 21 days after sulfide amendment, these mesocosms were producing almost no CH_4_. Further supporting our hypothesis, sulfide amendment showed no impact on methylotrophic methanogenesis.

Genes associated with sulfur assimilation were present in the genomes of both methylotrophic methanogen genera (*Methanomethylovorans* and *Methanolobus*) but were absent in the acetoclastic genomes. Sulfur assimilation can help mitigate sulfide toxicity by converting intracellular free sulfide into organic sulfur compounds and amino acids such as cysteine, thereby reducing the accumulation of toxic sulfide within the cell. Despite the methylotrophic methanogens analyzed here lacking some of the genes necessary for sulfur assimilation, methanogens are known to possess diverse mechanisms for sulfur uptake and assimilation, enabling some species to survive in high sulfide environments [61–64]. Across this study, the expression of sulfur assimilation genes by methylotrophic methanogens was generally low, with the exception of the PAPS reductase gene (*cysH*), which *Methanomethylovorans* expressed at higher levels in the methanol-amended P8 mesocosms. The presence and expression of these genes by methylotrophic, but not acetoclastic, methanogens suggest differences in their ability to cope with sulfide stress. However, targeted studies focusing on sulfur assimilation pathways and genes—particularly *cysH*—across diverse methanogenic metabolisms are necessary to fully understand the role of sulfur assimilation in sulfide tolerance.

To better understand how methylotrophic methanogens persist under such inhibitory conditions, we examined gene expression patterns in key methanogenic taxa. Despite *Methanomethylovorans* expressing methanogenic genes at similar levels in P7 and P8 sediments, total *Methanomethylovorans* gene expression was almost 10 times greater in P8. This suggests that other metabolic or physiological processes were critical to *Methanomethylovorans* activity, potentially associated with counteracting the stress imposed by high sulfide concentrations in P8 pore fluids. Sulfide toxicity is known to disrupt methanogenesis in both engineered and natural systems; however, the exact mechanisms remain poorly understood [65–68]. Sulfide toxicity occurs across many different microbial taxa and domains of life, most commonly by binding to and inhibiting cytochrome C oxidase, thereby disrupting the electron transport chain [69]. However, many methanogens, including *Methanothrix*, lack cytochromes, making this an unlikely mechanism of sulfide toxicity in these PPR wetland methanogens [70]. Alternative toxicity mechanisms include oxidative stress, DNA damage, membrane damage, and inactivation of redox centers in metalloenzymes [71–73].

In *Methanomethylovorans,* we observed significant upregulation of several genes associated with oxidative stress, DNA damage repair, and membrane damage in methanol-amended P8 mesocosms. One method of membrane damage could be through lipid peroxidation, which occurs when free radicals degrade membrane lipids, compromising cell integrity [74]. A lysophospholipid acyltransferase was the highest expressed gene across all genomes in this study. This enzyme plays an important role in phospholipid remodeling by incorporating fatty acid chains into membrane lipids [75], a strategy employed by some microbes for mitigating environmental stress [76–78]. High expression of this gene suggests that *Methanomethylovorans* actively repairs membrane damage caused by oxidative stress and/or lipid degradation. Beyond stress response genes, we also observed an increased expression in housekeeping genes, likely reflecting the increased cellular effort required to maintain homeostasis under sulfide-induced stress. Altogether, *Methanomethylovorans* combats sulfide toxicity by remodeling its membrane, repairing DNA damage, and upregulating stress response genes, enabling it to maintain cellular integrity to survive in sulfide-rich environments.

The inhibition of acetoclastic, but not methylotrophic, methanogenesis under high sulfide concentrations is likely related to Gibbs free energy yields associated with each metabolism. Methanogenesis from methanol generates significantly more energy (methyl-dismutating: ΔG°′ = −87.3 kJ/mol CH4; hydrogen dependent methylotrophy: ΔG°′ = −113.7 kJ/mol CH4) compared to acetate (ΔG°′ = −11 kJ/mol CH_4_) [21]. Such energy yields from methylotrophic methanogenesis could potentially allow *Methanomethylovorans* to expend energy counteracting sulfide toxicity, as indicated by increased expression of stress response and cellular maintenance genes. Similar energetic constraints are thought to control methanogenesis shifts from acteoclastic to hydrogenotrophic pathways under high ammonia conditions, partly due to the increased energy required to maintain osmotic balance under ammonia stress [79]. In this analogous instance, shifts from acetoclastic to methylotrophic methanogenesis may be expected because energy yields from acetoclastic methanogenesis are likely insufficient to support the cellular maintenance needed to counteract environmental stresses, such as sulfide.

## Conclusion

Here, we investigated the role of labile carbon substrates in supporting methanogenic metabolisms in surficial wetland sediments. Although this study focused on sediments from the PPR—one of the largest wetland complexes globally—the processes investigated here likely occur widely across saturated soils in both freshwater and saline environments. In mesocosm experiments where sediments were amended with different substrates, methylotrophic methanogenesis emerged as the dominant CH_4_-generating pathway, consistently contributing to high CH_4_ production rates. The functioning of this pathway across two distinct PPR wetlands is likely linked to the higher free energy available from this reaction, allowing investment in stress tolerance strategies including membrane remodeling and DNA damage repair to counteract sulfide toxicity. Further research is needed to understand if this phenomenon is limited to *Methylomethylovorans* or if it is widely observed across methylotrophic methanogens. We hypothesize that analogous processes may occur in other saturated environments, such as coastal wetlands affected by saltwater intrusion and elevated sulfide, emphasizing the need to integrate methylotrophic methanogenesis and the biogeochemical role of sulfide toxicity into models for improved predictions of wetland CH_4_ emissions.

## Supplementary Material

Bechtold_SI_wraf196

Supplemental_Data_1_wraf196

Supplemental_Data_2_wraf196

Supplemental_Data_3_wraf196

## Data Availability

The metatranscriptomic reads and 16S rRNA gene sequencing reads generated for and utilized in this manuscript have been deposited in the National Center for Biotechnology Information (NBCI) PRJNA1007388. Additionally, all MAGs and their DRAM v1.3.2 annotations and the Newick trees for phylogenomic and phylogenetic analyses, and a curated list of methanogens and methanotrophs used in this paper were from a previous publication and are available on Zenodo (http://dx.doi.org/10.5281/zenodo.10822869) [49].
