# Supplementary material for: Sulfide stress tolerance as a controller of methane production in temperate wetlands
Source: ISME J. 2025 Aug 28;19(1):wraf196. doi: 10.1093/ismejo/wraf196 (PMC12448728; doi:10.1093/ismejo/wraf196)
Supplement: Bechtold_SI_wraf196 [file bechtold_si_wraf196.docx]

Supporting Information for

**Sulfide stress tolerance as a controller of methane production in temperate wetlands**

Emily K. Bechtold (0000-0001-5092-6264)1, Jared B. Ellenbogen (0000-0003-1692-9044)^1^, Danhui Xin (0000-0002-5267-9727)2,a, Maricia Pacheco(0000-0003-1010-5095)3,4, Brandy M. Toner (0000-0002-3681-3455)3, Yu-Ping Chin (0000-0003-1427-9156)2, William A. Arnold (0000-0003-0814-5469)4, Sheel Bansal(0000-0003-1233-1707)^5^, and Michael J. Wilkins (0000-0002-3595-0853)1*

1Department of Soil and Crop Sciences, Colorado State University, Fort Collins, CO, 80523, USA

2Department of Civil, Construction, and Environmental Engineering, University of Delaware, Newark, DE, 19716, USA

3Department of Soil, Water, and Climate, University of Minnesota, MN, 55455, USA

4Department of Civil, Environmental, and Geo- Engineering, University of Minnesota, Minneapolis, MN, 55455, USA

^5^U. S. Geological Survey, Northern Prairie Wildlife Research Center, Jamestown, ND, 5840, USA

**a**Current Affiliation: Southern California Coastal Water Research Project Authority, Costa Mesa, CA, 92626, USA

*Corresponding Author: Michael J. Wilkins

Email: [Mike.Wilkins@colostate.edu](mailto:Mike.Wilkins@colostate.edu)

1170 Campus Delivery

Fort Collins, CO 80523

**Supplemental Figures**

Supplemental Figure 1. Relative abundance (%) of the methanogen community from field samples from two high methane emitting wetlands (P7 and P8) in the Prairie Pothole Region of North America. P7 generally has considerably lower pore water aqueous sulfide concentrations relative to P8. These sediment samples were then used in the mesocosm experiment described in this study.

Supplemental Figure 2. Temporal 16S rRNA gene data from the edge and open water eco-zones of each wetland of two wetlands, P7 and P8, in the Prairie Pothole Region (PPR) of North America. (A & B) The relative abundance of methanogens in P7 open-water and edge mesocosms shows that acetoclast relative abundance increase over time in response to acetate amendment. No change was observed in the methanogen community in response to formate, while methanol amendment resulted in enrichment of the methylotrophic methanogen, *Methanolobus.* (C & D) Methanogens in P8 mesocosms had a lower relative abundance and showed different responses to substrate amendment than in the P7 samples. Acetate and formate amendment did not enrich any methanogens in the P8 communities but amendment with methanol did result in an increased abundance of the methylotrophic methanogen, *Methanomethylovorans*. (E & F) Substrate amendment with all 3 substrates resulted in an increase in CH_4_ production in P7 mesocosms. CH_4_ production was measured using gas chromatography (GC). (G & H) The corresponding GC data in P8 shows that methanol amendment has the greatest impact on CH_4_ production. Additionally, acetate amendment and formate amendment had a smaller impact in P8 than in P7. Methane data represents the total CH_4_ accumulation over each 10 day period per mesocosm.

Supplemental Figure 3. Average CH_4_ production for each treatment (n=4) compared between P7 and P8 mesocosms containing sediment and water from the respective P7 and P8 wetland in the Prairie Pothole Region of North America. Each point represents CH_4_ produced over a 10 day period. Relationships between sites were analyzed for significance using a one-way analysis of variance test followed by a Tukey’s honest significant difference to determine pairwise differences.

 Supplemental Figure 4. Average aqueous sulfide concentration at the beginning and end of the experiment for each treatment and site (n=4 mesocosms each). The boxes represent the interquartile rage with the median sulfide concentration of each mesocosm given by the black line within each box.


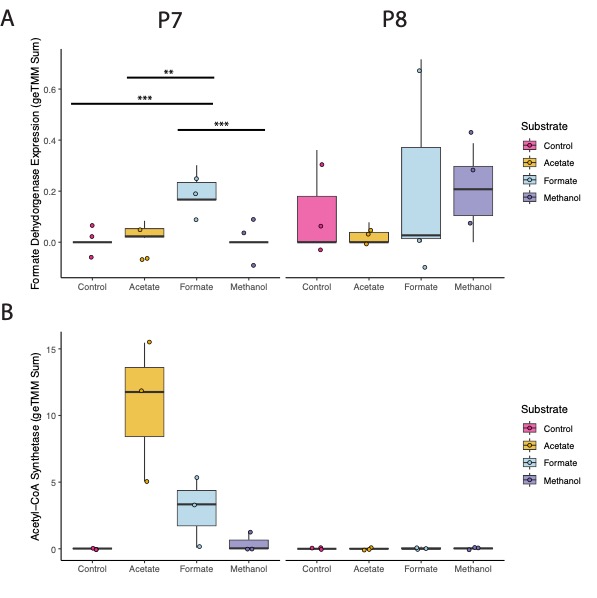


Supplemental Figure 5. (A) Average expression of the formate dehydrogenase gene from *UBA467* metagenome assembled genomes (MAGs) at the end of the experiment for each treatment and each site (n=3 metatranscriptomes). (B) (A) Average expression of the acetyl-CoA synthetase gene from *Methanothrix* MAGs at the end of the experiment for each treatment and each site (n=3 metatranscriptomes) shows an increase in expression in P7 acetate and formate amended mesocosms. The boxes represent the interquartile rage with the median expression based on geTMM (gene length corrected trimmed mean of M-values) normalization of each mesocosm given by the black line within each box. Mesocosms contained sediment and water from either the P7 or P8 wetland in the Prairie Pothole Region of North America.

Supplemental Figure 6. Differentially expressed genes in P7 mesocosms between the unamended control (left of each panel) and (A) acetate amended, (B) formate amended, and (C) methanol amended mesocosms (n = 3 metatranscriptomes). All three showed increased expression of genes associated with methane production. Differential expression was determined using MaAsLin2, and only genes with KEGG Orthology annotations were retained so to allow functional categorization via DRAM.

Supplemental Figure 7. Differentially expressed genes in P8 mesocosms between the unamended control and (A) acetate amended, (B) formate amended, and (C) methanol amended mesocosms (n = 3 metatranscriptomes). Acetate amendment showed very few differences in differential expression while formate amendment showed no differences. Methanol amended mesocosms showed increased expression in 2,329 genes of which 766 had DRAM annotations. 92 were related to methane production and 327 were categorized as Cell Structure/Function indicating that a large difference in the treatments was related to general cell maintenance and methanogenesis. Differential expression was determined using MaAsLin2, and only genes with KEGG Orthology annotations were retained so to allow functional categorization via DRAM.

Supplemental Figure 8. (A) Average expression of the assimilatory phosphoadenosine phosphosulfate (PAPS) reductase (*cysH*) gene from *Methanolobus* and *Methanomethylovorans* MAGs at the end of the experiment for each treatment and each site (n=3 metatranscriptomes) shows a significant increase in expression in *Methanomethylovorans* in the P8 methanol amended mesocosms.


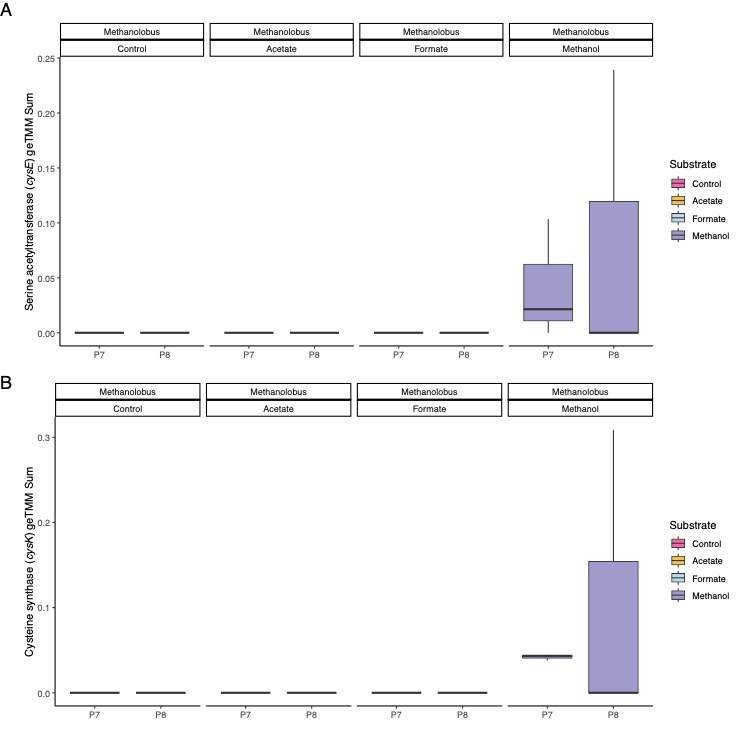


Supplemental Figure 9 (A) Average expression of the serine acetyltransferase (cysE) gene from *Methanolobus* MAGs at the end of the experiment for each treatment and each site (n=3 metatranscriptomes). (B) (A) Average expression of the cysteine synthase (cysK) gene from *Methanolobus* MAGs at the end of the experiment for each treatment and each site (n=3 metatranscriptomes) The boxes represent the interquartile rage with the median expression based on geTMM normalization of each mesocosm given by the black line within each box.

Supplemental Tables 1. Metagenomic samples and their corresponding NCBI accession number and sequencing depth.

| Sample | NCBI Accession | Depth (Gb) |
| --- | --- | --- |
| PPR_1022_P7D_M_E_Control2_Day30 | SAMN40344533 | 16.4 |
| PPR_1022_P7D_M_E_Formate3_Day30 | SAMN40344534 | 14.6 |
| PPR_1022_P7D_M_E_Methanol3_Day30 | SAMN40344535 | 17.1 |
| PPR_1022_P7S_M_E_Acetate3_Day30 | SAMN40344536 | 12.5 |
| PPR_1022_P8D_M_E_Acetate2_Day30 | SAMN40344537 | 18.5 |
| PPR_1022_P8D_M_E_Control4_Day30 | SAMN40344538 | 16.8 |
| PPR_1022_P8D_M_E_Formate2_Day30 | SAMN40344539 | 15.2 |
| PPR_1022_P8D_M_E_Methanol2_Day30 | SAMN40344540 | 14.4 |
| PPR_1022_P8S_M_E_Acetate3_Day30 | SAMN40344541 | 15.6 |

Supplemental Table 2. Metatranscriptomic samples and their corresponding NCBI accession number and sequencing depth.

| Sample | NCBI Accession | Depth (Gb) |
| --- | --- | --- |
| PPR_1022_P7_Deep_Acetate_1_37_day30 | SAMN47991929 | 14.5 |
| PPR_1022_P7_Deep_Acetate_2_38_day30 | SAMN47991930 | 13.2 |
| PPR_1022_P7_Deep_Acetate_3_39_day30 | SAMN47991931 | 13.5 |
| PPR_1022_P7_Deep_Control_1_5_day30 | SAMN47991932 | 14.9 |
| PPR_1022_P7_Deep_Control_2_6_day30 | SAMN47991933 | 17.3 |
| PPR_1022_P7_Deep_Control_3_7_day30 | SAMN47991934 | 17.4 |
| PPR_1022_P7_Deep_Formate_1_53_day30 | SAMN47991935 | 15.2 |
| PPR_1022_P7_Deep_Formate_2_54_day30 | SAMN47991936 | 13.5 |
| PPR_1022_P7_Deep_Formate_3_55_day30 | SAMN47991937 | 5.6 |
| PPR_1022_P7_Deep_Methanol_1_21_day30 | SAMN47991938 | 13.7 |
| PPR_1022_P7_Deep_Methanol_2_22_day30 | SAMN47991939 | 13.1 |
| PPR_1022_P7_Deep_Methanol_3_23_day30 | SAMN47991940 | 19.6 |
| PPR_1022_P8_Deep_Acetate_1_45_day30 | SAMN47991941 | 8.8 |
| PPR_1022_P8_Deep_Acetate_2_46_day30 | SAMN47991942 | 8.3 |
| PPR_1022_P8_Deep_Acetate_3_47_day30 | SAMN47991943 | 2.7 |
| PPR_1022_P8_Deep_Control_1_13_day30 | SAMN47991944 | 12.3 |
| PPR_1022_P8_Deep_Control_2_14_day30 | SAMN47991945 | 11.4 |
| PPR_1022_P8_Deep_Control_3_15_day30 | SAMN47991946 | 10.2 |
| PPR_1022_P8_Deep_Formate_1_61_day30 | SAMN47991947 | 12.4 |
| PPR_1022_P8_Deep_Formate_2_62_day30 | SAMN47991948 | 17.8 |
| PPR_1022_P8_Deep_Formate_3_63_day30 | SAMN47991949 | 12.1 |
| PPR_1022_P8_Deep_Methanol_1_29_day30 | SAMN47991950 | 9.0 |
| PPR_1022_P8_Deep_Methanol_2_30_day30 | SAMN47991951 | 13.2 |
| PPR_1022_P8_Deep_Methanol_3_31_day30 | SAMN47991952 | 12.2 |
